# Earthworms Significantly Alter the Composition, Diversity, Abundance and Pathogen Load of Fungal Communities in Sewage Sludge from Different Urban Wastewater Treatment Plants

**DOI:** 10.3390/pathogens14050409

**Published:** 2025-04-24

**Authors:** Manuel Aira, Ana Gómez-Roel, Jorge Domínguez

**Affiliations:** Grupo de Ecoloxía Animal (GEA), Universidade de Vigo, E-36310 Vigo, Spain; ana.gomez.roel@uvigo.gal (A.G.-R.); jdguez@uvigo.gal (J.D.)

**Keywords:** earthworms, fungal diversity, fungal pathogens, sewage sludge, vermicomposting

## Abstract

Management of sewage sludge is of ongoing concern because this waste product is generated continuously and contains high levels of harmful constituents. Among these constituents, fungal pathogens are of increasing concern. Vermicomposting can reduce the amounts of bacterial pathogens in sewage sludge; however, information about the effects of earthworms on fungal pathogens is limited or non-existent. We therefore aimed to determine whether vermicomposting can control fungal pathogens present in sewage sludge. Using next-generation sequencing techniques, we characterized fungal communities in sewage sludge from eight wastewater treatment plants (WWTPs) and in casts (feces) of earthworms feeding on sewage sludge. Fungal communities in earthworm casts primarily included taxa that were absent from sewage sludges, indicating a significant change in fungal composition. Changes in fungal diversity depended on the source of sewage sludge (WWTP). All of the sewage sludges contained low levels of fungal pathogens, most of which were significantly reduced or eliminated by earthworms, such as *Armillaria*, *Cystobasidium*, *Exophiala* and *Ophiosthoma*. Moreover, earthworm gut transit enhanced beneficial (saprotrophic) fungi like *Arthrobotrys*, *Aseroe*, *Crepidotus* and *Trichurus*. Overall, digestion of sewage sludge by earthworms alone generated a mainly pathogen-free fungal community with a high proportion of saprotrophic taxa, which would enhance nutrient cycling rates.

## 1. Introduction

The increase in urbanization has resulted in a corresponding increase in consumption of resources and, as a result, the continuous, ever-increasing production of sewage sludge in wastewater treatment plants (WWTPs). This situation raises significant environmental concerns due to the presence of hazardous contaminants and pathogens, including those considered in legislation, such as heavy metals and *Escherichia coli* and *Salmonella* spp. In addition, there is a growing list of emerging contaminants, such as pharmaceuticals [1], poly-fluoroalkyl substances [2] and a wide range of human pathogens [3]. The management of sewage sludge has thus emerged as a key issue in environmental policies that favor recycling over land disposal. Nevertheless, a high proportion of sewage sludge, estimated to be about 50% (Available online: http://ec.europa.eu/eurostat, accessed on 1 February 2025), is still disposed of on land, creating new environmental risks, such as an increase in antibiotic resistance genes [4].

The high numbers of pathogens in sewage sludge, including viruses, bacteria, protozoa, fungi and helminths [5], are of primary concern. Nevertheless, most research conducted to date has concentrated on bacteria and viruses [5], probably because these are included in legislation in numerous countries [6]. Consequently, much research has focused on characterizing bacterial communities, including both pathogenic and non-pathogenic strains [3]. Despite the emphasis placed by the World Health Organization (WHO) on various fungal human pathogens [7] and the diversity and amounts of fungal pathogens in sewage sludge [8], research characterizing fungal communities and pathogens remains limited. Furthermore, the use of antifungal drugs, together with the excessive application of fungicides in agriculture and animal husbandry, has contributed to an increase in antifungal resistance and has also facilitated the dissemination of resistance in the environment [9].

Vermicomposting has proven to be an easily implementable technology that can transform sewage sludge into a valuable organic fertilizer [10,11]. Furthermore, the vermicomposting process has been shown to be effective against human pathogens, including viruses, bacteria and helminths [12]. However, research concerning the effects of vermicomposting on fungal pathogens remains limited. The vermicomposting process consists of two stages. The earthworms first digest the waste and produce casts (feces), which then undergo a maturation process, resulting in vermicompost [10]. A few studies have outlined the changes that occur in fungal communities during the vermicomposting process [10,13,14,15]. However, only Gómez-Roel et al. [16] concentrated on fungal pathogens, reporting that vermicomposting was able to remove parasitic and pathogenic fungal taxa. Previous studies have demonstrated that earthworms can eliminate up to 99% of the fungal taxa found in sludge [10,16], indicating that this effect may contribute to reducing and/or removing pathogenic fungi from sewage sludge.

However, it is not clear whether the reduction in fungal pathogens during vermicomposting, as reported by Gómez-Roel et al. [16], is a general outcome of the process or if it varies depending on the fungal species and/or the WWTP that is the source of the sludge. We used high-throughput internal transcribed spacer (ITS) rRNA gene sequencing, together with metataxonomic analysis, with two primary objectives. First, we determined how earthworm digestion affects the composition and diversity of fungal communities in sewage sludge samples from different wastewater treatment plants. Second, we characterized the pathogenic fungi present in various sludge samples and examined their fate after transit through the earthworm gut. We hypothesized that, as most fungal taxa seem to be eliminated during earthworm digestion, fungal pathogens should be either eradicated or their numbers significantly reduced.

## 2. Materials and Methods

### 2.1. Sewage Sludge and Sampling of Earthworm Casts

We set up earthworm cultures and fed them with fresh sewage sludge obtained from WWTPs in Moaña (19,458 inhabitants), Burela (9580), Miño (6056), Ordes (12,589), Cerceda (5085), Caldas de Reis (9834) and Vilagarcía de Arousa (37,761), and a WWTP associated with a dairy factory (InLeit Ingredients SLU). All of the companies are located in Galicia (northwestern Spain). The main physicochemical and microbiological characteristics of the sewage sludge used are shown in Supplementary Table S1. The eight sewage sludge samples were characterized by a pH ranging from slightly acidic to basic (mean: 7.1, range 5.8–8.1), highly variable electrical conductivity (mean: 328, range 67–1168 µS cm^−1^) and high moisture (mean: 82%, range 71–89%) and organic matter (mean: 72%, range 60–80%) contents. The total carbon contents (mean: 33%, range 29–37%) and nitrogen values (mean: 5.5%, range 2.6–7.8%) were relatively consistent across all the WWTPs, with the exception of the Cerceda WWTP, which had the lowest values (Supplementary Table S1). Concerning microbiological characteristics, most sewage sludge exhibited high levels of microbial biomass (mean: 39,341 µg C g^−1^ dw, range 4950–89,024 µg C g^−1^ dw), associated with high microbial activity (mean: 1555 µg CO_2_ g^−1^ dw h^−1^, range 293–3782 µg CO_2_ g^−1^ dw h^−1^), except for those from the Cerceda WWTP (Supplementary Table S1).

We removed mature earthworms from the cultures to sample their fresh casts. For this purpose, we washed the earthworms and then placed them in sterile Petri dishes (five dishes with 20 individuals each) in an incubator chamber for 24 h to allow them to void their guts [10]. We then sampled the casts under sterile conditions and stored them in sterile Eppendorf tubes at −80 °C. We took five samples for each combination of sewage sludge and earthworm casts for all WWTPs, except for the Moaña WWTP, where we took fifteen samples.

### 2.2. Amplification, Sequencing and Analysis of the ITS Region of rRNA Genes

We extracted DNA from sewage sludge and cast samples (0.25 g fresh weight) in a laminar flow hood to prevent contamination, using the MO-BIO PowerSoil^®^ kit (QIAGEN, Madrid, Spain) according to the manufacturer’s protocol. The ITS RNA gene was sequenced using the primers ITS1f (CTTGGTCATTTAGAGGAAGTAA) and ITS2 (GCTGCGTTCTTCATCGATGC) in a 2 × 250 Illumina MiSeq run. Amplicon sequence variants (ASVs) were inferred using DADA2 (version 1.34.0). ASVs were used as they provide more accurate and reproducible results than operative taxonomic units (OTUs) [17,18]. Raw sequences lacking primers and barcodes were processed according to the DADA2 pipeline (Available online: https://benjjneb.github.io/dada2/ITS_workflow.html (accessed on 12 January 2025)). ITS forward/reverse read pairs were filtered using standard filtering parameters (maxN = 0, truncQ = 2, rm.phix = TRUE and maxEE = 2). ASVs were independently inferred from the forward and reverse reads of each sample using the run-specific error rates, and the read pairs were then merged. One set made only from forward reads was retained. Chimeras were identified in each sample, and ASVs were removed if they were classified as chimeric in a sufficient proportion of the samples in which they appeared. The taxonomy of ASVs was inferred using the RDP naive Bayesian classifier [19,20] in comparison with the UNITE database (version 10) with a bootstrap confidence level of 80. ASVs that remained unclassified at the phylum level were excluded from further analysis. We used the ASVs derived from forward sequences as these included had more ASVs (9000) and sequences (3,269,686) than those inferred from paired reads (5393 ASVs and 3,184,129 sequences). The sequence data were deposited in the GenBank SRA database under accession number PRJNA 1,230,886.

### 2.3. Bioinformatic and Statistical Analysis

All the data were analyzed and plotted using the phyloseq [21], tidyverse [22], patchwork [23], vegan [24], ComplexHeatmap [25], rstatix [26], ggh4x [27] and microeco [28] packages implemented in R version 4.4.0 [29].

To study the effect of earthworm gut transit on the abundance of fungal phyla, genera and ASVs in sewage sludge across WWTPs, we used the DESeq2 package [30], as previously described [10]. We performed this analysis on a prevalence-filtered dataset that removed 84% of fungal ASVs, which accounted for 6% of the total sequences.

We estimated the proportion of fungal ASVs of earthworm origin (native), i.e., those fungal ASVs present in earthworm casts but not in sewage sludge samples. For this purpose, we divided the data into eight sets for each WWTP and rarefied them. We then determined the native fungal ASVs within each WWTP as those that were not shared between the sludge and earthworm cast samples. We modeled the effect of WWTP on the proportion of native ASVs using generalized linear models (GLM function, binomial family) and conducted Tukey post-hoc tests using the multcomp library [31]. Additionally, the impact of the sludge fungal community composition on native fungal ASVs was estimated by comparing the ASVs found in the casts from each WWTP (pairs, and three-, four-, five-, six-, seven- and eight-wise) and identifying the shared ASVs. A high level of shared taxa among casts from different WWTPs would suggest a dominant role of sewage sludge in the composition of native fungal ASVs. We used one-way ANOVA to examine the number of shared taxa across levels of comparison (pair-wise to eight-way) and then conducted post-hoc comparisons with the false discovery rate (FDR)-corrected *t*-test. Taxonomic alpha diversity was calculated as the number of observed ASVs, while diversity was assessed using the inverse Simpson index. The raw values were multiplied by 1000, and the mean value was calculated for each diversity index. The impact of earthworm gut transit on both the richness and diversity of sewage sludge across wastewater treatment plants (WWTPs) and sample types (sewage sludge and earthworm castings) was examined using ANOVA followed by a paired *t*-test (anova_test and *t*-test in the rstatix package). The *p* values obtained in the *t*-tests were adjusted using the FDR method. Taxonomic beta diversity at the ASV level was measured by the differences in the composition of the bacterial community between samples of sewage sludge and earthworm casts across wastewater treatment plants. Differences in beta diversity were determined by considering ASV abundance (Bray–Curtis) or not (Jaccard) using permutational multivariate analysis of variance (PERMANOVA) on the filtered ASV table (variance-stabilized transformation). We conducted pairwise PERMANOVA tests to examine differences between sewage sludge and earthworm casts across WWTPs, adjusting *p*-values using the FDR method. We used PCoA to illustrate changes in beta diversity.

To determine the ecological roles of fungi in sewage sludge and earthworm casts, we used the FUNGuildR database [32], accessed on February 2025 [33], a tool that assigns trait information by mapping it to a taxonomic classification. We only included ASVs rated as ‘probable’ and ‘highly probable’ in the analysis (Supplementary Table S2). To conduct a more detailed analysis of the fate of fungal pathogens, we focused only on ASVs classified as “Pathotroph” with a ‘highly probable’ confidence rating. The ASVs considered were from *Cystobasidium slooffiae*, *Cy. lysinophilum*, *Cy. Pinicola* and *Chalciporus piperatus*. In addition, we searched for fungal pathogens using the EuPathDB database (Available online: https://benlangmead.github.io/aws-indexes/k2 (accessed on 12 January 2025), based on https://veupathdb.org/veupathdb/app (accessed on 12 January 2025)). We found that only *Candida parapsilosis*, *Aspergillus carbonarius*, *Exophiala mesophila*, *Malassezia restricta* and *M. globosa* were present in the samples. We also searched our analysis for the species and genera of fungal pathogens included in the WHO report [7], nominally *Acremonium* spp., *Aspergillus fumigatus*, *Candida auris*, *C. albicans*, *C. tropicalis*, *C. parapsilopsis*, *Coccidioides* spp., *Cryptococcus gattii*, *Cr. neoformans*, *Curvularia lunata*, *Falciformispora senegalensis*, *Fusarium* spp., *Histoplasma* spp., *Lomentospora prolificans*, *Madurella* spp., *Mucorales*, *Nakaseomyces glabrata* (*Candida glabrata*), *Paracoccidioides* spp., *Pichia kudriavzeveii* (*Candida krusei*), *Pneumocystis jirovecii*, *Scedosporium* spp., *Talaromyces marneffei* and *Zopfia rosatii.* We also searched for the presence of the fungal pathogen genera *Aspergillus*, *Blastomyces*, *Candida*, *Cryptococcus*, *Pneumocystis* and *Mucormycetes*, noted in the report *One Health: Fungal Pathogens of Humans, Animals, and Plants* (Available online: https://www.ncbi.nlm.nih.gov/books/NBK549988/ (accessed on 12 January 2025)). We also searched for the presence of fungal pathogen genera identified by Assress et al. [34], which include *Afipia*, *Ceratocystis*, *Cladophialophora*, *Cladosporium*, *Cuniculitrema*, *Fonsecaea*, *Lacazia*, *Mucor*, *Mycosphaerella*, *Ochroconis*, *Olpidium*, *Paecilomyces*, *Penicillium*, *Phyllosticta*, *Phymatotrichopsis*, *Puccinia*, *Rhodotorula*, *Sporothrix*, *Synchytrium*, *Talaromyces*, *Trichosporon* and *Ustilago.* We concluded with a list of 40 genera of fungal pathogens, including 115 species of fungal pathogens, along with another 23 species of fungal pathogens as defined above. The impact of earthworm gut transit on the abundance of various fungal guilds was analyzed using the same statistical model applied for alpha diversity.

## 3. Results

### 3.1. Impact of Earthworm Gut Transit on the Composition of Fungal Communities in Sewage Sludge

**Figure 1:**
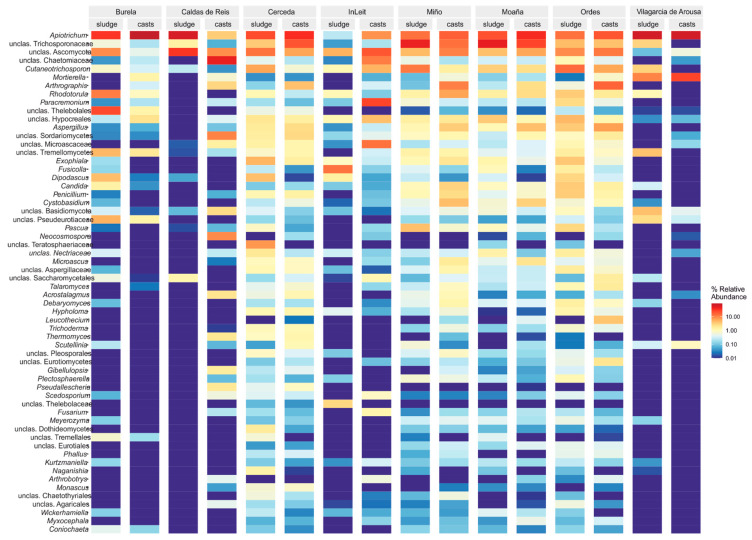
Heatmap of the 60 most abundant fungal genera found in sewage sludge from eight WWTPs, as well as after transit through the gut of the earthworm *Eisenia andrei*. The mean values for both sewage sludge and earthworm casts are displayed.

The fungal communities in sewage sludge primarily consisted of fungi included in the Ascomycota and Basidiomycota phyla, although their abundance varied significantly between WWTPs (Table 1). Thus, Ascomycota was the dominant phylum in some cases (the Burela, Cerceda, InLeit and Ordes WWTPs), while Basidiomycota dominated in sludge from other WWTPs. Other fungal phyla were present in the sludge, including Aphelidiomycota, Chytridiomycota, Entorrhizomycota and Mortierellomycota; however, the abundance of these phyla was generally low (<1%), except for Mortierellomycota in the sludge from the Vilagarcía de Arousa WWTP (Table 1). Earthworm gut transit significantly increased the abundance of the fungal phylum Mortierellomycota in the sewage sludge from the Burela, Caldas de Reis, InLeit and Vilagarcía de Arousa WWTPs (Wald test, Supplementary Table S3). Conversely, earthworm gut transit significantly reduced the abundance of Ascomycota (Burela and InLeit WWTPs) and Basidiomycota (Caldas de Reis, Miño and Moaña WWTPs), while it increased the abundance of these in the sludge from the Vilagarcía de Arousa WWTP (Wald test, Supplementary Table S3). At the genus level, the differences in composition of the sludge from the different WWTPs became clearer, as did the impact of earthworm gut transit (Figure 1). Thus, earthworm gut transit consistently increased the abundance of *Arthrographis*, *Leucothecium* and *Microascus* in sewage sludge from all WWTPs (Figure 1, Wald test, Supplementary Table S4). Conversely, earthworm gut transit significantly reduced the abundance of the genera *Cryptococcus* and *Dipodascus* in sludge from several WWTPs. Several fungal genera underwent changes in abundance depending on the source of the sewage sludge WWTP, including *Cutaneotrichosporon*, *Pascua* and *Apiotrichum* (Figure 1, Wald test, Supplementary Table S4). These results were extended by analyzing the data at the ASV level, as different ASVs were identified for the same fungal genera (Wald test, Supplementary Table S5).

**Figure 2:**
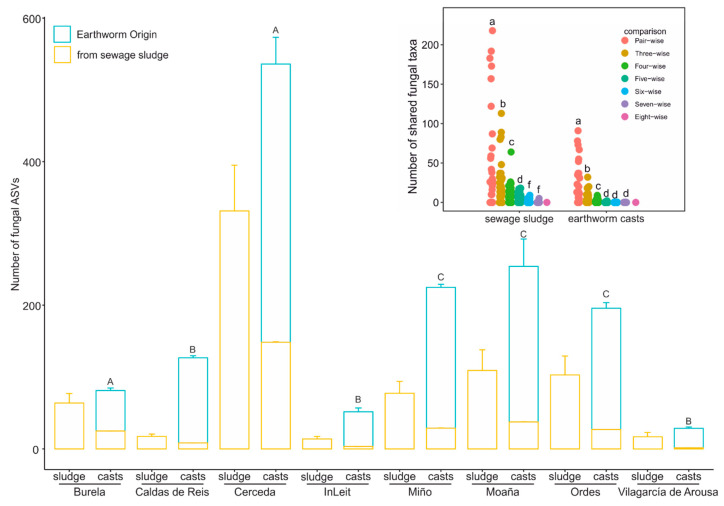
Source of fungal ASVs in fungal communities in sewage sludge before and after earthworm gut transit. Fungal ASVs are categorized as ‘earthworm origin’ if they are present in the earthworm cast and absent from the corresponding sewage sludge. Different letters in the main plot indicate significant differences in the proportion of fungal ASVs among earthworm casts (Tukey HSD test). The inset shows the number of ASVs of earthworm origin shared by earthworm casts among WWTPs in paired, triple, quadruple and five-, six-, seven- and eight-way comparisons, as well as for the sewage sludges from the eight WWTPs. A high degree of shared ASVs among casts from different WWTPs would indicate a dominant role of sewage sludge in the composition of native fungal ASVs. Different letters in the inset plot indicate significant differences in the number of shared native fungal ASVs among comparisons.

Most fungal ASVs in the earthworm casts were native, meaning they were not found in the corresponding sludge samples (Figure 2). Furthermore, the quantity of native fungal ASVs differed among the WWTPs (glm, *p* < 0.0001), grouped into three based on the proportion of native fungal ASVs, based on the Tukey HSD post-hoc test (Figure 2). These groups were formed by the sewage sludge from Burela and Cerceda (approximately 70%), Miño, Moaña and Ordes (around 85%) and Caldas de Reis, InLeit and Vilagarcía de Arousa (about 94% native fungal ASVs, as shown in Figure 2). Furthermore, there were no shared native fungal ASVs among the earthworm casts generated from the sewage sludge from the eight WWTPs, indicating that source of the sewage sludge influenced the composition of native fungal ASVs (Figure 2 insert). Consequently, the number of shared ASVs among earthworm casts generated from the different sewage sludge samples decreased significantly (ANOVA, *p* < 0.0001) from pair-wise to eight-wise comparisons (Figure 2 insert). Furthermore, this decline was already evident in the mean number of shared ASVs in three-wise comparisons, which was significantly lower than the number found in two-wise comparisons (Figure 2 insert). The mean number of shared taxa in four- and five-wise comparisons was below one, reaching zero in six-, seven- and eight-wise comparisons.

### 3.2. Impact of Passage Through the Earthworm Gut on the Diversity of Fungal Communities in Sewage Sludge

**Figure 3:**
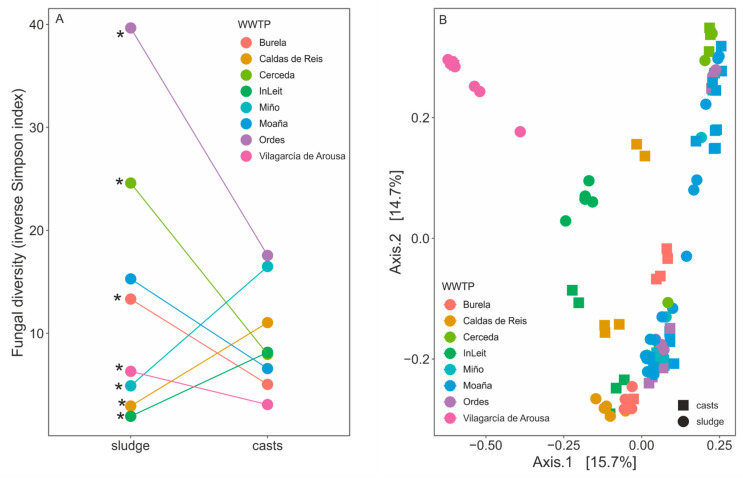
Changes in fungal diversity of sewage sludge from several WWTPs before and after passage through the gut of the earthworm species *Eisenia andrei* are shown as (**A**) alpha diversity estimated using the inverse Simpson index and (**B**) beta diversity determined by principal coordinate analysis of Bray–Curtis distances. Asterisks in panel A indicate significant differences between sewage sludge and earthworm casts within each WWTP (paired *t*-test, FDR-corrected).

The effect of earthworm gut transit on the fungal alpha diversity of sewage sludge depended on the WWTP (interaction WWTP × type, *p* < 0.0001, Figure 3A). In some instances, fungal diversity decreased significantly (Burela, Cerceda, Ordes and Vilagarcía de Arousa, paired *t*-test), increased (Caldas de Reis, InLeit and Miño, paired *t*-test), or no effect was observed (Moaña, paired *t*-test, Figure 3A). These changes were also apparent when examining fungal richness, with significant decreases (Burela and Cerceda) or increases (Caldas de Reis, InLeit and Miño) in fungal richness following earthworm gut transit (Supplementary Figure S1A). However, there was no interaction between WWTP and sample type (*p* = 0.17). Earthworm gut transit significantly altered the beta diversity, whether fungal abundance was considered (Bray–Curtis distances, Figure 3B) or not (Jaccard distances, Supplementary Figure S1B). In both instances, the impact of earthworm gut transit significantly depended on the WWTP (interaction WWTP × type, *p* = 0.001 for both distances), as demonstrated by varying patterns of separation between sewage sludge and casts across WWTPs. Post-hoc pairwise PERMANOVAS revealed that most sludge–earthworm cast comparisons within each WWTP were significant (*p* < 0.05, Figure 3B, Supplementary Figure S1B), except for those of Cerceda and Miño for both distances (Bray–Curtis and Jaccard).

### 3.3. Effect of Earthworm Gut Transit on Fungal Guilds and Pathogens

**Figure 4:**
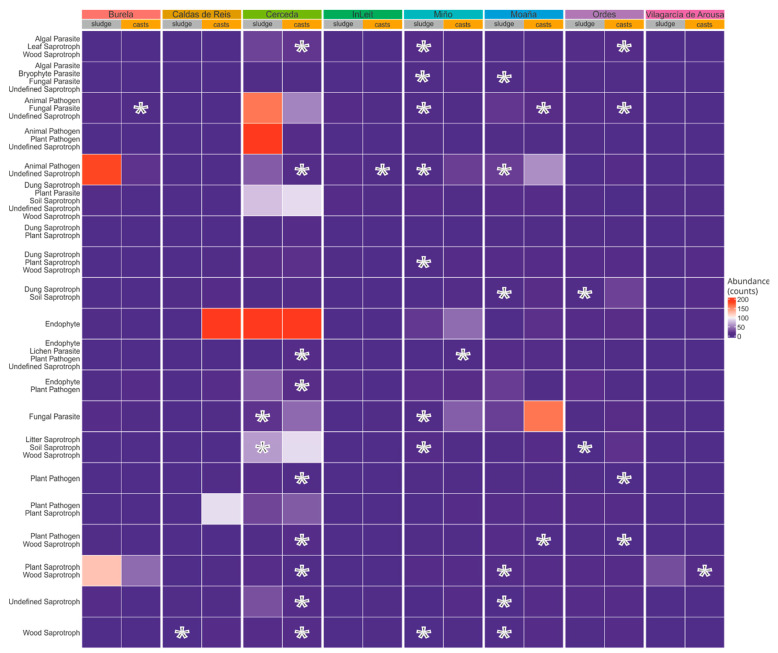
Heatmap of high-abundance fungal guilds present in sewage sludge and earthworm casts from different WWTPs. The mean abundance in sequence counts is shown. Asterisks indicate significant differences between sewage sludge and fresh earthworm casts (paired *t*-test, FDR-corrected). The position of the asterisks indicates either a decrease (located in casts) or an increase after passing through the earthworm gut (located in sludge).

Only 24% of the ASVs were able to be assigned to a functional guild (2204 out of 9000), accounting for 11% of the total sequences. The most abundant fungal guilds included “Animal Pathogen–Undefined Saprotroph”, “Animal Pathogen–Fungal Parasite–Undefined Saprotroph”, “Fungal Parasite” and “Animal Pathogen–Plant Pathogen–Undefined Saprotroph”, among others (Figure 4). The impact of earthworm gut transit on the abundance of fungal guilds was significantly influenced by the WWTP (interaction of WWTP × sample type, Supplementary Table S5) for several of the guilds. For instance, earthworm gut transit had a significant effect on the abundance of the “Animal Pathogens–Undefined Saprotrophs”, “Plant Saprotrophs–Wood Saprotrophs” and “Wood Saprotrophs” guilds in the sewage sludge from the different WWTPs (Figure 3, paired *t*-test, Supplementary Table S6). Specifically, earthworm gut transit increased the abundance of “Animal Pathogen–Undefined Saprotroph” in samples from the Miño and Moaña WWTPs, while it decreased it in those from the Cerceda and InLeit WWTPs (paired *t*-test, Figure 4). Earthworm gut transit consistently increased the abundance of the “Litter Saprotroph–Soil Saprotroph–Wood Saprotroph”, “Dung Saprotroph–Soil Saprotroph” and “Fungal Parasites” guilds in several STPs (paired *t*-test, Figure 4). There was no common trend for the effects of earthworms on guilds containing the term “pathogen”. For example, in some guilds, particularly those including “Animal Pathogen”, earthworm gut transit either increased or decreased abundance depending on the WWTP, such as in the guilds “Animal Pathogen–Fungal Parasite–Undefined Saprotroph” or “Animal Pathogen–Undefined Saprotroph” (paired *t*-test, Figure 4). In other instances, particularly for guilds including “Plant Pathogen”, earthworm gut transit significantly decreased their abundance in the sewage sludge from several WWTPs (paired *t*-test, Figure 4). The main fungal genera involved in these shifts were *Armillaria*, *Cystobasidium*, *Exophiala*, *Ophiosthoma* and *Rhodotorula* among the pathogens, and *Arthrobotrys*, *Aseroe*, *Crepidotus*, *Phallus* and *Trichurus* among the saprotrophs (Supplementary Table S6).

We identified thirteen fungal ASVs from five genera (*Mucor*, *Rhizomucor*, *Rhizopus*, *Lichtheimia* and *Circinella*) within the order Mucorales. The abundance of all of these genera was relatively low, with fewer than 10 sequences obtained for most of them (Supplementary Table S7). A total of nine species of fungal pathogens were identified: *Aspergillus carbonarius*, *Candida parapsilosis*, *Chalciporus piperatus*, *Cystobasidium lysinophilum*, *Cy. pinicola*, *Cy. slooffiae*, *Exophiala mesophila*, *Malassezia globosa* and *M. restricta* (Supplementary Table S6). The abundance of these pathogens was low, except for *C. parapsilosis* and *Cy. slooffiae*. Earthworms significantly reduced the abundance of *C. parapsilosis* in the sewage sludge from the Burela WWTP while increasing it in the sewage sludge from the Miño WWTP, similar to the observed effect on *Cy. slooffiae* in the sewage sludge from the Miño WWTP (paired *t*-test, Supplementary Table S7).

We identified twenty-one genera of fungal pathogens, and similar to the fungal pathogen species, most were present in low abundance (<100 sequences, Supplementary Table S7). Despite the variable range of abundance, most fungal pathogen genera were prevalent in sewage sludge samples from the majority of WWTPs (Table 2, Supplementary Table S7). The impact of earthworm gut transit on the abundance of the primary nine genera depended on the fungal pathogen and the source of the sewage sludge (WWTP) (Table 2). For example, the abundance of *Aspergillus* was significantly higher in the earthworm casts from sewage sludge from some WWTPs than in those from others, which was also true for the genera *Candida*, *Penicillium* and *Talaromyces* (Table 2, paired *t*-test, Supplementary Table S7). By contrast, earthworm gut transit significantly removed *Cryptococcus* and *Trichosporon* in just one WWTP, although the prevailing trend was for a decrease or removal of these phyla across all WWTPs (Table 2, paired *t*-test, Supplementary Table S7). Finally, the impact of earthworms on the abundance of the genera *Fusarium*, *Rhodotorula* and *Scedosporium* varied by WWTP, and notable increases and decreases were observed (Table 2, paired *t*-test).

## 4. Discussion

Fungal communities varied significantly in the sewage sludge samples from the different WWTPs, although the fungal diversity and richness are comparable to those reported in earlier studies [10,16,34,35]. The study findings showed that simple passage through the gut of earthworms significantly altered the composition and diversity of fungal communities in the sewage sludge samples from eight wastewater treatment plants (WWTPs). These findings confirm and extend existing studies on the impact of earthworms on fungal communities [10,16]. Regarding the composition of the fungal communities, most fungal taxa identified in earthworm casts were native, consistent with previous reports [10,16]. The prevalence of native fungal taxa in earthworm casts appears to be a common finding, as it has also been noted for earthworms in mineral soils [36]. Notably, there was minimal overlapping in the composition of native fungal ASVs among different WWTPs, indicating that sewage sludge significantly influences the composition of these microbial communities. Additionally, the number of native fungal taxa, while significantly increased, demonstrated considerable variation among WWTPs. It is important to note that the physicochemical and microbiological parameters of sewage sludge appeared unrelated to this phenomenon, as their values fluctuated across WWTPs but remained consistent across the three groups examined concerning the proportion of native fungi (Burela–Cerceda, Miño–Moaña–Ordes and Caldas de Reis–InLeit–Vilagarcía de Arousa). Future studies should seek to identify what factors may be contributing to this phenomenon. For non-native fungal taxa, i.e., fungal ASVs found in the sewage samples and capable of passing through the earthworm gut, the impact of earthworms differed depending on the specific WWTP considered. For some fungal ASVs, earthworm gut transit significantly increased the abundance of *Dipodascus* and decreased that of *Debaryomyces* across all WWTPs. By contrast, for others like *Pascua* and *Cutaneotrichosporon*, the abundance varied depending on the WWTP where the sewage sludge was obtained. The changes in composition had a significant effect on fungal alpha diversity. Interestingly, earthworm gut transit either decreased or increased fungal diversity depending on the source of the sludge (WWTP), an effect that seems to be related to the initial fungal diversity of sewage sludge. Previous studies have also shown this variable effect, reporting increases [10] and decreases [16]. The changes in fungal alpha diversity, along with alterations in fungal composition, led to significant shifts in beta diversity, as previously reported [10,16]. The changes in beta diversity were also dependent on the source of the sample (WWTP). This observation highlights the influence of the source of the sewage sludge on the earthworm-derived effects and may stem from variations in the number of native fungal ASVs, as well as the varying patterns of alpha diversity in the different sewage sludge samples.

Fungal guilds varied significantly among the different sewage sludges, mainly consisting of saprotrophs with small contributions from pathogens and parasites, as previously reported [16]. A general trend was observed in the abundance of saprotrophic guilds increasing after earthworm gut transit, in contrast to previous findings [10]. Along with the expected microbial succession related to the aging of earthworm casts during vermicomposting [10,16], this would result in vermicompost containing fungal communities that facilitate decomposition. Application of the vermicompost as an organic amendment would thus enhance soil nutrient cycling. It should be noted that the guild assignments from FUNGuild may not be very precise. For instance, a fungus categorized in the “Fungal Parasite–Plant Saprotroph” guild could belong to any of these guilds, making it impossible to determine specifically whether it is a saprotroph or a parasite.

The abundance of fungal pathogens in the sewage sludges was significantly lower than reported in other studies, in which levels of up to 8% relative abundance was reported for some fungal taxa [34]. Therefore, the relative abundance of most of the fungal pathogens detected was well below 1. This may be attributed to the efficacy of wastewater treatment processes, which are known to reduce fungal pathogen loads [34,35,36,37], or to the fact that sewage sludge from small urban wastewater treatment plants (such as those in the present study) tends to contain lower amounts of pathogens [3,38], or a combination of both factors. However, earthworm gut transit successfully reduced or even eliminated most of the fungal pathogens detected, as previously reported [16], indicating a sanitizing effect. This may be attributed to the known antifungal activity present in earthworm gut fluids, which could selectively inhibit the growth of ingested fungi [39], or it may simply reflect the digestive capacity of earthworms [40]. This suppressive effect, observed against fungi of the genus *Fusarium*, was also documented in soils, where earthworms significantly reduced the abundance of *F. oxysporum* [41]. Furthermore, this suppressive effect may extend from casts to vermicompost, which is recognized for its antifungal activity against various plant pathogenic fungi, including *Rhizoctonia solani*, *Alternaria solani* and *Aspergillus niger*, among others [42].

However, certain fungal pathogens, including those from the genera *Aspergillus*, *Candida*, *Fusarium* and *Penicillium*, were found to be more abundant in earthworm casts than in sludge. This phenomenon appears to be common, as it was also observed in different earthworm species (*Eudrilus eugeniae*, *Lampito mauritii*, *Eisenia fetida* and *Perionyx excavatus*) that were fed on soil or cow dung for pathogenic fungi from the genera *Aspergillus*, *Mucor* and *Cladosporium*, among others [43]. This could be problematic as earthworms may serve as vectors for these pathogens. One possible reason for the increase in pathogens in earthworms could be the impact of the continued contact with the sewage sludge. For instance, several studies have indicated that various contaminants, such as pesticides [44], pharmaceuticals [45] and microplastics [46], significantly elevate stress levels in earthworms and consequently affect their health status [47]. This would increase the susceptibility of earthworms to infections caused by these predominantly opportunistic pathogens [48].

## 5. Conclusions

In this study, we used amplicon sequencing of the ITS rRNA gene, which may have limitations in taxonomic resolution at the species level. We also utilized FUNGuildR to determine the trophic mode of fungi found in sewage sludge and earthworm castings. While this protocol has inherent limitations due to its predictive nature, we attempted to address these for fungal pathogens in two steps. First, we were very strict in delimiting fungal taxonomy, using bootstrapping values higher than 80%; and second, we sought to identify fungi that were clearly established as pathogenic in three databases and/or reports. Earthworm gut transit significantly altered the fungal communities in sewage sludge, mainly because the fungal communities in earthworm casts predominantly consist of fungi not found in sewage sludge. Further studies may include more resolving techniques such as qPCR to determine the abundance of critical fungal pathogens. Furthermore, the impact of earthworms on fungal alpha diversity, whether leading to an increase or decrease, depended on the specific sewage sludge. The fungi mainly found in sewage sludge belonged to saprotrophic and, to a lesser extent, pathogenic and parasitic guilds. Earthworm gut transit reduced the pathogenic and parasitic fungi while mainly enhancing the saprotrophic fungi, thus boosting the degradation capacity of the fungal communities. We detected low numbers of fungal pathogens in the sewage sludges and found that the earthworms were capable of reducing or removing most pathogenic fungi, with a few minor exceptions. The fact that most pathogenic and non-pathogenic fungi from sewage sludge were unable to thrive in the earthworm gut, and that this effect was dependent on sewage sludge, could be attributed to several factors, including (i) selective digestion by earthworms, (ii) the presence of antagonistic fungi among the native earthworm fungi and (iii) the presence of bacteria capable of suppressing pathogenic fungi (not analyzed in this dataset). Further studies should determine the mechanisms responsible for this effect.

## Figures and Tables

**Table 1:** Relative abundance of fungal phyla found in sludge from eight wastewater treatment plants and after passing through the gut of the earthworm *Eisenia andrei*. Mean and standard errors.

		Burela	Caldas de Reis	Cerceda	InLeit	Miño	Moaña	Ordes	Vilagarcía de Arousa
Aphelidiomycota	sludge	0 ± 0	0 ± 0	0.004 ± 0.002	0 ± 0	0 ± 0	0.002 ± 0.002	0.014 ± 0.014	0 ± 0
	casts	0 ± 0	0 ± 0	0 ± 0	0 ± 0	0 ± 0	0 ± 0	0 ± 0	0 ± 0
Ascomycota	sludge	48.08 ± 6.85	34.68 ± 3.75	56.62 ± 1.91	95.72 ± 0.60	20.12 ± 1.99	26.08 ± 6.08	52.80 ± 1.34	7.69 ± 2.79
	casts	6.64 ± 0.17	92.454 ± 1.42	36.89 ± 1.36	88.11 ± 4.60	49.56 ± 5.29	23.6 ± 2.13	62.01 ± 2.26	2.64 ± 0.22
Basidiobolomycota	sludge	0 ± 0	0 ± 0	0 ± 0	0 ± 0	0 ± 0	0 ± 0	0 ± 0	0 ± 0
	casts	0 ± 0	0 ± 0	0 ± 0	0 ± 0	0.01 ± 0.01	0 ± 0	0 ± 0	0 ± 0
Basidiomycota	sludge	51.91 ± 6.85	65.31 ± 3.75	43.16 ± 1.93	3.93 ± 0.55	79.70 ± 2.01	73.63 ± 6.08	46.97 ± 1.30	85.68 ± 2.95
	casts	91.97 ± 0.11	6.83 ± 1.4	62.94 ± 1.36	11.40 ± 4.58	49.45 ± 5.32	76.12 ± 2.16	36.63 ± 2.21	70.247 ± 1.69
Blastocladiomycota	sludge	0 ± 0	0 ± 0	0 ± 0	0.33 ± 0.15	0 ± 0	0 ± 0	0 ± 0	0 ± 0
	casts	0 ± 0	0.002 ± 0.002	0 ± 0	0 ± 0	0 ± 0	0 ± 0	0 ± 0	0 ± 0
Chytridiomycota	sludge	0 ± 0	0 ± 0	0.012 ± 0.002	0 ± 0	0 ± 0	0.004 ± 0.003	0 ± 0	0 ± 0
	casts	0 ± 0	0 ± 0	0.002 ± 0.002	0 ± 0	0 ± 0	0 ± 0	0 ± 0	0 ± 0
Entorrhizomycota	sludge	0 ± 0	0 ± 0	0.001 ± 0.001	0 ± 0	0 ± 0	0 ± 0	0 ± 0	0 ± 0
	casts	0 ± 0	0 ± 0	0 ± 0	0 ± 0	0 ± 0	0 ± 0	0 ± 0	0 ± 0
Kickxellomycota	sludge	0 ± 0	0 ± 0	0 ± 0	0 ± 0	0 ± 0	0 ± 0	0 ± 0	0.024 ± 0.024
	casts	0 ± 0	0 ± 0	0 ± 0	0 ± 0	0 ± 0	0 ± 0	0 ± 0	0 ± 0
Monoblepharomycota	sludge	0 ± 0	0 ± 0	0 ± 0	0 ± 0	0 ± 0	0.001 ± 0.001	0 ± 0	0 ± 0
	casts	0 ± 0	0 ± 0	0 ± 0	0 ± 0	0 ± 0	0 ± 0	0 ± 0	0 ± 0
Mortierellomycota	sludge	0.002 ± 0.002	0 ± 0	0.067 ± 0.01	0 ± 0	0.14 ± 0.02	0.25 ± 0.10	0.12 ± 0.03	6.52 ± 3.79
	casts	1.38 ± 0.07	0.705 ± 0.09	0.05 ± 0.01	0.47 ± 0.14	0.83 ± 0.10	0.20 ± 0.03	1.29 ± 0.06	27.10 ± 1.55
Mucoromycota	sludge	0 ± 0	0 ± 0	0.003 ± 0.002	0 ± 0	0 ± 0	0 ± 0	0 ± 0	0.08 ± 0.04
	casts	0 ± 0	0.002 ± 0.001	0.005 ± 0.004	0 ± 0	0 ± 0	0.001 ± 0.001	0 ± 0	0.002 ± 0.002
Neocallimastigomycota	sludge	0 ± 0	0 ± 0	0 ± 0	0 ± 0	0 ± 0	0 ± 0	0 ± 0	0 ± 0
	casts	0 ± 0	0 ± 0	0.003 ± 0.003	0 ± 0	0 ± 0	0 ± 0	0 ± 0	0 ± 0
Olpidiomycota	sludge	0 ± 0	0 ± 0	0 ± 0	0 ± 0	0.006 ± 0.006	0.002 ± 0.002	0 ± 0	0 ± 0
	casts	0 ± 0	0 ± 0	0 ± 0	0 ± 0	0 ± 0	0 ± 0	0 ± 0	0 ± 0
Rozellomycota	sludge	0.001 ± 0.001	0 ± 0	0.12 ± 0.02	0 ± 0	0.02 ± 0.01	0.03 ± 0.01	0.08 ± 0.03	0 ± 0
	casts	0 ± 0	0 ± 0	0.10 ± 0.02	0 ± 0	0.14 ± 0.03	0.02 ± 0.01	0.06 ± 0.01	0 ± 0
Zoopagomycota	sludge	0 ± 0	0 ± 0	0 ± 0	0 ± 0	0 ± 0	0 ± 0	0 ± 0	0 ± 0
	casts	0 ± 0	0.002 ± 0.002	0 ± 0	0 ± 0	0 ± 0	0 ± 0	0 ± 0	0 ± 0

**Table 2:** Main fungal pathogen genera found in sludge from eight wastewater treatment plants and after passing through the gut of the earthworm *Eisenia andrei*. Asterisks indicate significant differences in abundance between sewage sludge and earthworm casts within each fungal pathogen genera and WWTP (paired *t*-test, FDR corrected *p*-values). Mean and standard errors of sequence counts.

		Burela	Caldas de Reis	Cerceda	InLeit	Miño	Moaña	Ordes	Vilagarcía de Arousa
*Aspergillus*	sludge	13 ± 4	0 ± 0 *	1225 ± 148	1.2 ± 1.2	71 ± 8 *	304 ± 112 *	157 ± 23 *	0 ± 0
	casts	28 ± 13	44 ± 9	1813 ± 359	7 ± 3	242 ± 14	2100 ± 751	845 ± 137	0 ± 0
*Candida*	sludge	545 ± 186	1.4 ± 1.4	77 ± 17	3 ± 3	74 ± 6 *	290 ± 110	176 ± 31	54 ± 28
	casts	18 ± 9	0 ± 0	107 ± 30	2 ± 1	242 ± 23	389 ± 160	181 ± 50	0.6 ± 0.6
*Cryptococcus*	sludge	1 ± 1	0 ± 0	208 ± 16 *	0 ± 0	10 ± 3	62 ± 24	12 ± 5	2 ± 1
	casts	0 ± 0	0 ± 0	0 ± 0	0 ± 0	1 ± 1	0 ± 0	0 ± 0	0 ± 0
*Fusarium*	sludge	0 ± 0	0 ± 0	113 ± 18 *	0 ± 0	3 ± 2 *	3 ± 2	8 ± 2	0 ± 0
	casts	0 ± 0	0 ± 0	0.4 ± 0.4	0 ± 0	12 ± 4	10 ± 5	5 ± 3	0 ± 0
*Penicillium*	sludge	5 ± 2	0 ± 0	365 ± 66	0 ± 0	53 ± 11 *	123 ± 44	148 ± 40	0 ± 0
	casts	0 ± 0	27 ± 9.	445 ± 95	4 ± 2	161 ± 23	255 ± 104	107 ± 19	0 ± 0
*Rhodotorula*	sludge	4291 ± 957 *	1 ± 1	759 ± 137 *	28 ± 6 *	112 ± 7 *	507 ± 195	177 ± 17	179 ± 77
	casts	410 ± 32	0 ± 0	188 ± 32	4 ± 2	536 ± 44	1307 ± 534	214 ± 45	0 ± 0
*Scedosporium*	sludge	24 ± 8 *	5 ± 2 *	514 ± 111 *	1.2 ± 1.2 *	8 ± 3 *	47 ± 10 *	52 ± 6 *	0 ± 0
	casts	1.2 ± 1.2	383 ± 55	278 ± 56	102 ± 15	15 ± 5	121 ± 16.231	68 ± 5	0 ± 0
*Talaromyces*	sludge	2 ± 1	0 ± 0	318 ± 66 *	0 ± 0	22 ± 43	71 ± 19	53 ± 10	0 ± 0
	casts	12 ± 10	2 ± 1	492 ± 70	5 ± 4	66 ± 14	170 ± 61	212 ± 57	0 ± 0
*Trichosporon*	sludge	45 ± 8 *	0 ± 0	7± 7	21 ± 16	2 ± 2	7 ± 4	1 ± 1	0 ± 0
	casts	0 ± 0	0 ± 0	0 ± 0	0 ± 0	0 ± 0	0 ± 0	0 ± 0	0 ± 0

## Data Availability

Sequences were uploaded to the GenBank SRA database under accession PRJNA1230886.

## Supplementary Materials

The following supporting information can be downloaded at: https://www.mdpi.com/article/10.3390/pathogens14050409/s1, Figure S1: Changes in fungal diversity of sewage sludge from several WWTPs before and after passage through the gut of the earthworm species *Eisenia andrei* are shown as (A) alpha diversity estimated as observed richness and (B) beta diversity determined by principal coordinate analysis of Jaccard distances. Asterisks in panel A indicate significant differences between sewage sludge and earthworm casts within each WWTP (paired *t*-test, FDR corrected); Table S1: Main physico-chemical characteristics of sewage sludge from eigth WWTPs (Mean and standard errors, N = 5); Table S2: FUNGuildR assigned trait information for fungal ASVs (amplicon sequence variants) from sewage sludge of different WWTPs and their corresponding earthworm casts from earthworm species *Eisenia andrei*; Table S3: Differential abundance in fungal phyla of sewage slugde from different WWTPs and casts of earthworm species Eisenia andrei analyzed using negative binomial models as implemented in the package DESeq2 [30]; Table S4: Differential abundance in fungal genera of sewage slugde from different WWTPs and casts of earthworm species *Eisenia andrei* analyzed using negative binomial models as implemented in the package DESeq2 [30]; Table S5: Differential abundance in fungal ASVs (amplicon sequence variants) of sewage slugde from different WWTPs and casts of earthworm species *Eisenia andrei* analyzed using negative binomial models as implemented in the package DESeq2 [30]; Table S6: Output from ANOVA analysis and post-hoc tests (paired *t*-test) on the effect of transit through the earthworm gut and WWTP on the abundance of main fungal guilds. Significant effects are marked in red; Table S7: Output from ANOVA analysis and abundance (mean and standard error of sequence counts) on the effect of transit through the earthworm gut and WWTP on the abundance of detected fungal pathogen genera and species.
