# Supplementary figures and images for: Earthworms Significantly Alter the Composition, Diversity, Abundance and Pathogen Load of Fungal Communities in Sewage Sludge from Different Urban Wastewater Treatment Plants
Source: Pathogens. 2025 Apr 24;14(5):409. doi: 10.3390/pathogens14050409 (PMC12114242; doi:10.3390/pathogens14050409)

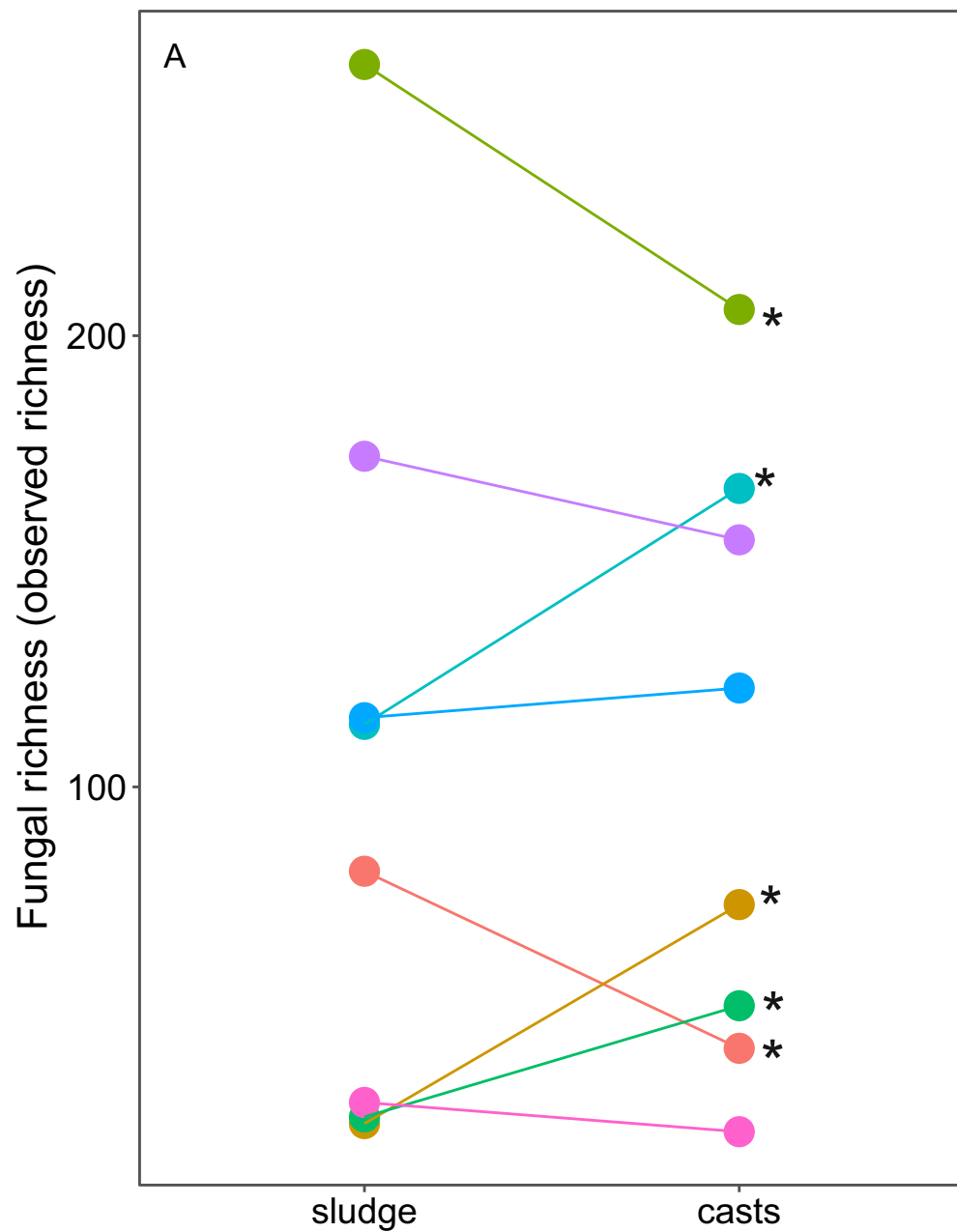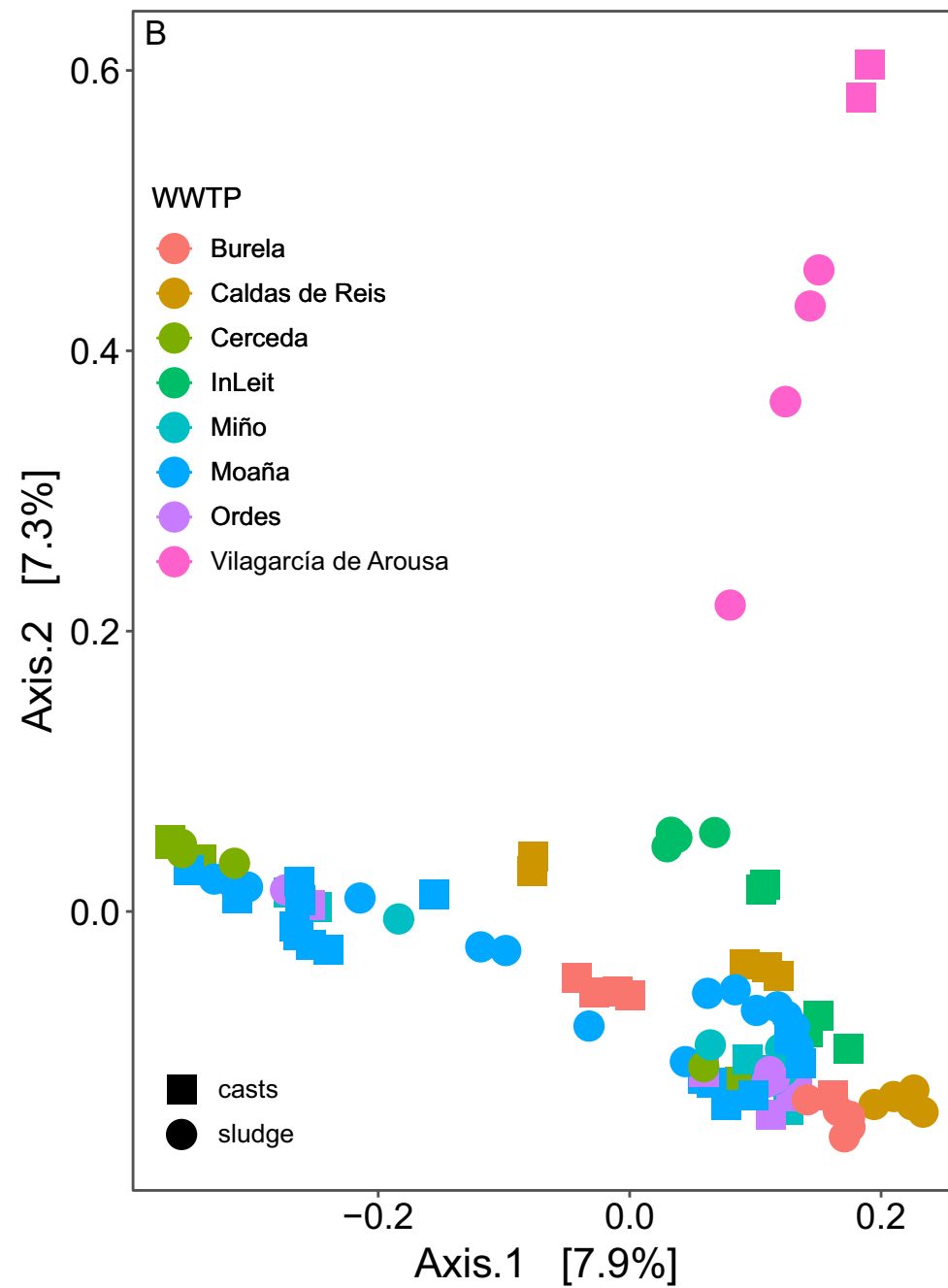

Supplement: Supplementary file 1 [file pathogens-14-00409-s001.zip › Supplementary Figure S1.pdf]
